# Supplementary material for: Combined Radiomodifying Effect of Fucoidan from the Brown Alga Saccharina cichorioides and Pacificusoside D from the Starfish Solaster pacificus in the Model of 3D Melanoma Cells
Source: Biomolecules. 2023 Feb 23;13(3):419. doi: 10.3390/biom13030419 (PMC10046073; doi:10.3390/biom13030419)
Supplement: Supplementary file 1 [file biomolecules-13-00419-s001.zip › biomolecules-2192762-supplementary.pdf]

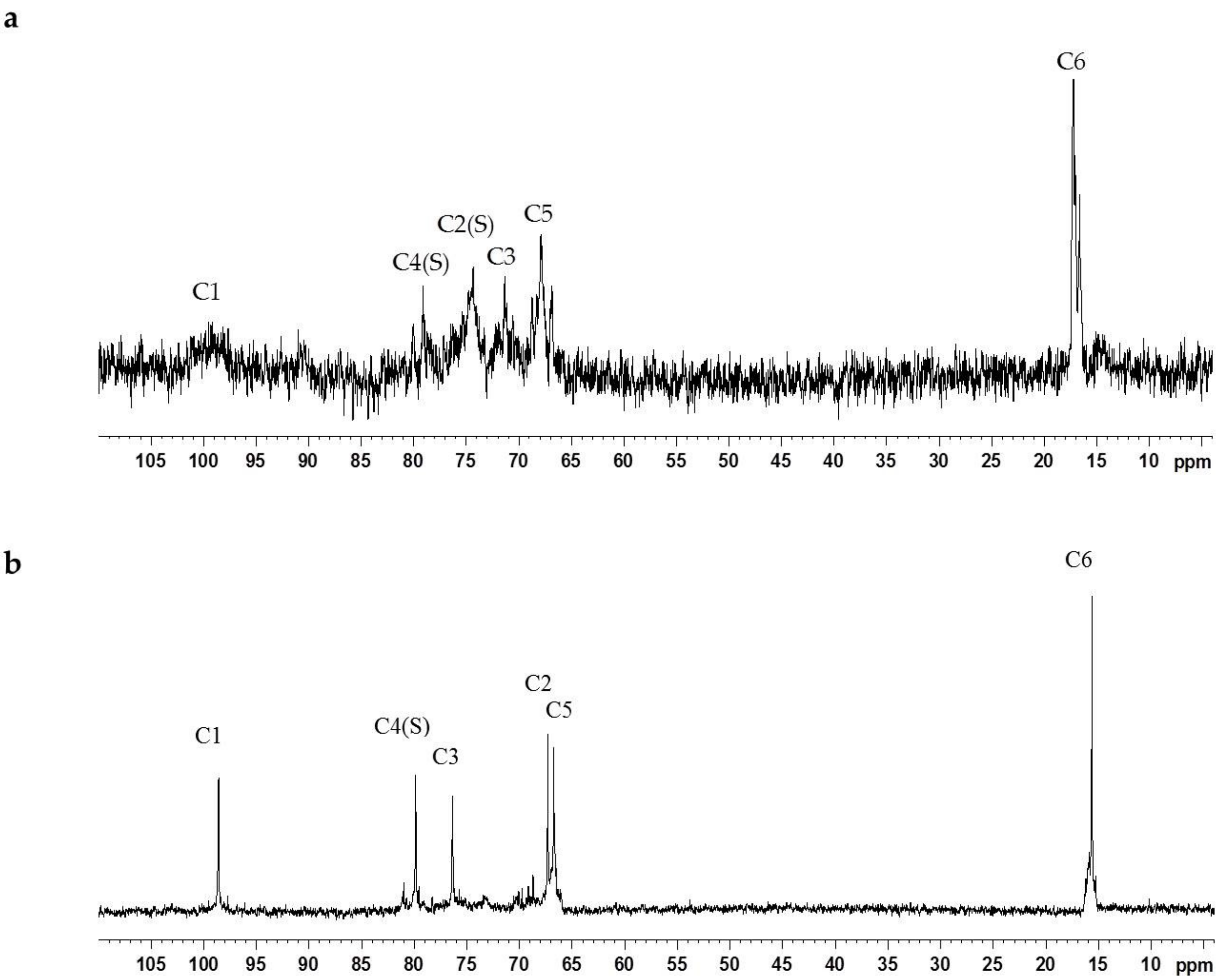

Supplementary Figure S1. ^{13}C NMR spectrum of (**a**) fucoidan ScF from *S. cichorioides* and *(***b***)* fucoidan derivative ScF_AH obtained by autohydrolysis. Signals (C1–C6) of α-(1→3)-L-fucopyranose residues of the main chain are indicated.

a

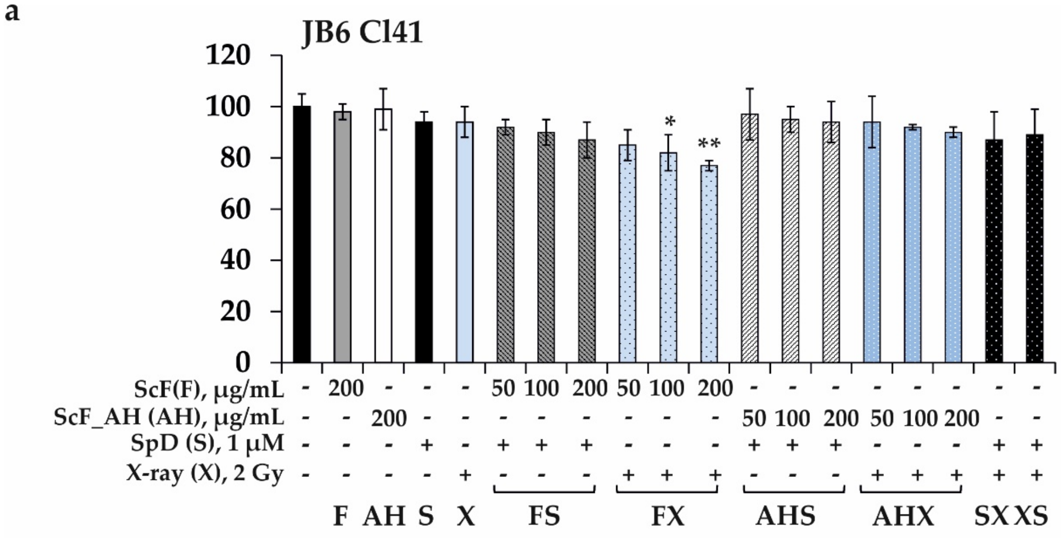

b

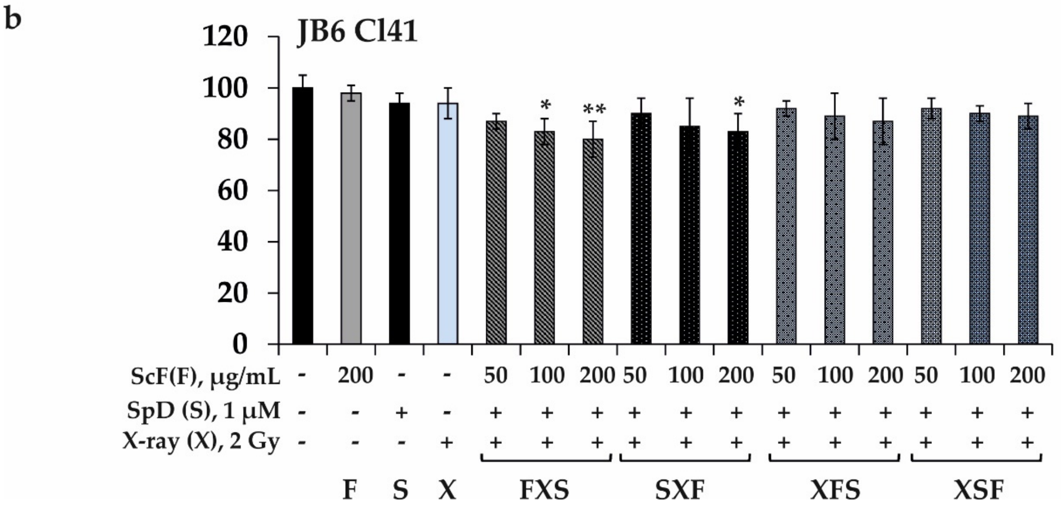

Supplementary Figure S2. Two-component (**a**) and three-component (**b**) combined effect of fucoidan from *S. cichorioides* (ScF), its derivative (ScF_AH) with pacificusoside D from *S. pacificus* (SpD) or X-ray on the viability of 3D normal epidermal cells JB6 Cl41.

JB6 Cl41 spheroids were treated by (**a**) ScF (50, 100, 200 µg/mL) in combination with SpD (1 µM) (FS combination); ScF (50, 100, 200 µg/mL) in combination with X-ray (FX combination); ScF_AH (50, 100, 200 µg/mL) in combination with SpD (1 µM) (AHS combination); ScF_AH (50, 100, 200 µg/mL) in combination with X-ray (AHX combination); SpD (1 µM) in combination with X-ray (2 Gy) (SX combination); X-ray (2 Gy) in combination with SpD (1 µM) (XS combination) and (**b**) ScF (50, 100, 200 µg/mL) in combination with X-ray (2 Gy) and SpD (1 µM) (FXS combination); SpD (1 µM) in combination with X-ray (2 Gy) and ScF (50, 100, 200 µg/mL) (SXF combination); X-ray (2 Gy) in combination with ScF (50, 100, 200 µg/mL) and SpD (1 µM) (XFS combination); and X-ray (2 Gy) in combination with SpD (1 µM) and ScF (50, 100, 200 µg/mL) (XSF combination) for total 72 h. The cell viability was estimated using the MTS assay. Data show the mean of three independent experiments ± SD. A one-way ANOVA and Tukey's HSD test for multiple comparisons indicated the statistical significance (*$p < 0.05$, **$p < 0.01$).
